# Antigenically Modified Human Pluripotent Stem Cells Generate Antigen-Presenting Dendritic Cells

**DOI:** 10.1038/srep15262

**Published:** 2015-10-16

**Authors:** Jieming Zeng, Chunxiao Wu, Shu Wang

**Affiliations:** 1 Institute of Bioengineering and Nanotechnology, Singapore; 2 Department of Biological Sciences, National University of Singapore, Singapore

## Abstract

Human pluripotent stem cells (hPSCs) provide a promising platform to produce dendritic cell (DC) vaccine. To streamline the production process, we investigated a unique antigen-loading strategy that suits this novel platform. Specifically, we stably modified hPSCs using tumour antigen genes in the form of a full-length tumour antigen gene or an artificial tumour antigen epitope-coding minigene. Such antigenically modified hPSCs were able to differentiate into tumour antigen-presenting DCs. Without conventional antigen-loading, DCs derived from the minigene-modified hPSCs were ready to prime a tumour antigen-specific T cell response and further expand these specific T cells in restimulation processes. These expanded tumour antigen-specific T cells were potent effectors with central memory or effector memory phenotype. Thus, we demonstrated that immunocompetent tumour antigen-loaded DCs can be directly generated from antigenically modified hPSCs. Using such strategy, we can completely eliminate the conventional antigen-loading step and significantly simplify the production of DC vaccine from hPSCs.

Dendritic cell (DC) vaccine is becoming a new therapeutic modality for cancer^1^,^2^. This therapeutic strategy exploits the power and specificity of the immune system to fight against cancer, yet avoids the devastating and life-threatening side effects of traditional cancer therapies. DC-based immunotherapy has a much better safety profile and may provide better quality of life for cancer patients. However, it remains challenging to prepare high-quality DC vaccines in large quantity to induce clinically significant anti-cancer immunity due to the complexities in making such living cell products^3^,^4^. Hence, a simplified manufacturing process is necessary to ultimately improve both the accessibility and therapeutic efficacy of DC vaccines^5^.

Currently, most DC vaccines are generated from patient blood cells^6^. A large amount of peripheral blood mononuclear cells (PBMCs) are collected from the patient via an invasive leukapheresis process. Monocytes are then isolated from PBMCs and further differentiated into DCs. These monocyte-derived DCs (moDCs) are loaded with tumour antigens and matured before injection into the patient. This production process is complicate and full of technical and logistic difficulties. The end products are costly as exemplified by Dendreon’s Provenge, the first ever FDA-approved DC-based vaccine for prostate cancer^7^. The qualities of such produced DC vaccines are highly variable due to unpredictable and uncontrollable patient-to-patient variation. With these inconsistent DC products, it is difficult to optimize those critical parameters that may further improve vaccine efficacy in clinical trials. Moreover, such patient blood cell-derived DC vaccines are often limited in supply, which makes it impossible to clinically evaluate the benefit of high dosage and frequent vaccination. All the above-mentioned issues are largely associated with the use of patient blood cells for DC vaccine production. To avoid these issues, it is imperative to employ an alternative platform that is reliable, standardizable and patient blood cell-independent. Naturally, in the age of pluripotency, human pluripotent stem cells (hPSCs) may well serve such a purpose^8^. As we have demonstrated earlier, hPSC-derived DCs (hPSC-DCs) are capable of presenting not only peptide antigen to antigen-specific CD8+ T cells^9^, but also glycolipid antigen to invariant natural killer T (iNKT) cells^10^. These proven functional capabilities of hPSC-DCs further validate the use of hPSCs to develop DC vaccines.

To produce DC vaccine, antigen-loading is a crucial step that defines the specificity of vaccine-induced anti-tumour immune response. Most commonly used antigen-loading approaches include peptide-pulsing, protein-loading, tumour lysate-loading, RNA/DNA transfection and viral transduction^11^. These conventional approaches require not only the production of various clinical-grade tumour antigen payloads, but also the unavoidable and sometimes detrimental cell manipulations to deliver the antigen payloads into DCs. Furthermore, in large-scale manufacturing, the antigen-loading step needs to be repeated for every batch of DC vaccine, which poses a great challenge to yield consistent products. Although these conventional approaches are also applicable to hPSC-DCs^9^,^10^, a simpler antigen-loading solution is highly desirable for making DC vaccine from hPSCs. To this end, we stably modified the hPSCs with tumour antigen genes in this study and demonstrated that such antigenically modified hPSCs were able to differentiate into functional tumour antigen-presenting DCs. Using this novel antigen-loading strategy, no conventional antigen-loading step is required for generating tumor antigen-presenting DCs from hPSCs, thus the production of hPSC-DC cancer vaccine can be significantly simplified.

## Results

### Tumour antigen gene-modified hPSCs produce tumour antigen-expressing DCs

**Figure 1:**
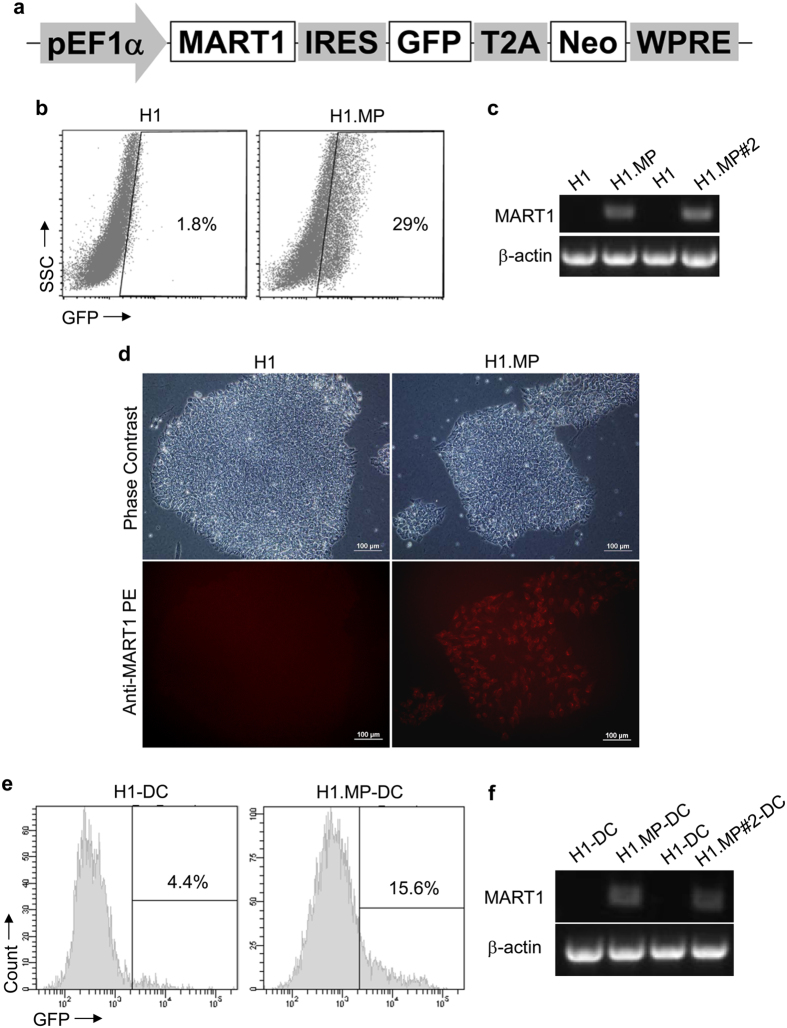
Tumour antigen gene-modified hPSCs produce tumour antigen-expressing DCs. (**a**) Structure of lentivector LV.MP carrying a tumour antigen gene *MART-1*. (**b**) GPF expression in H1.MP cells, a H1 cell line generated by LV.MP transduction and G418 selection, as detected by flow cytometry. (**c,d**) *MART-1* expression in H1.MP cells as measured by RT-PCR (**c**) and immunostaining (**d**). (**e**) GFP expression in H1.MP-derived DCs (H1.MP-DCs) as detected by flow cytometry. (**f**) *MART-1* expression in H1.MP-DCs as measured by RT-PCR.

To investigate whether hPSCs can be modified by tumour antigen gene and subsequently used to derive tumour antigen-expressing DCs, we generated a lentivector carrying a *MART-1* gene, designated as LV.MP (Fig. 1a). LV.MP was also containing a *GFP* gene as reporter and a neomycin-resistance gene for drug selection (Fig. 1a). This lentivector was used to transduce an hPSC line, H1. After selection with G418, G418-resistent H1 lines were generated. One of these lines, H1.MP showed substantial GFP expression (Fig. 1b). Moreover, *MART-1* expression was also observed in H1.MP as demonstrated at both RNA level (Fig. 1c) and protein level (Fig. 1d). Both H1.MP line and parental H1 line were then used to generate DCs, designated as H1.MP-DCs and H1-DCs, respectively. Although both *GFP* and *MART-1* were still expressed in H1.MP-DCs, the expression levels were low (Fig. 1e,f). These results indicate that it is feasible to derive tumour antigen-expressing DCs from tumour antigen gene-modified hPSCs. However, a further increase of tumour antigen expression level in these DCs is necessary for sufficient antigen presentation on DC surface.

### DCs derived from tumour antigen gene-modified hPSCs present tumour antigen

**Figure 2:**
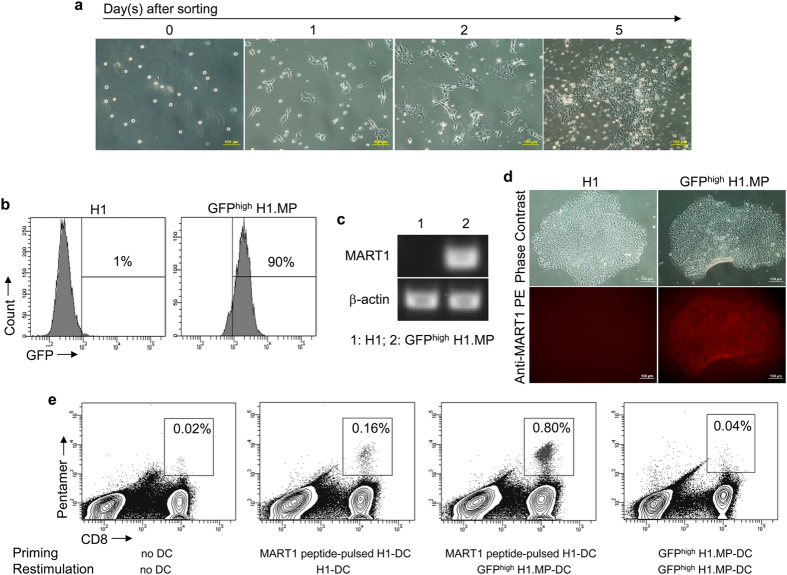
DCs derived from tumour antigen gene-modified hPSCs present tumour antigen. (**a**) Proliferation of GFP^high^ H1.MP cells after sorting. (**b**) GFP expression in sorted GFP^high^ H1.MP cells as detected by flow cytometry. (**c,d**) *MART-1* expression in GFP^high^ H1.MP cells as measured by RT-PCR (**c**) and immunostaining (**d**). (**e**) Expansion of primed MART-1-specific CD8+ T cells by GFP^high^ H1.MP-derived DCs (GFP^high^ H1.MP-DCs) as detected by pentamer staining and flow cytometry.

To have higher level of tumour antigen expression in hPSC-DCs, GFP^high^ H1.MP cells were enriched by fluorescence-activated cell sorting. These GFP^high^ H1.MP cells survived the cell sorting process as demonstrated by cell proliferation after sorting (Fig. 2a). The resulting H1 cell line not only showed a high percentage of GFP+ cells (Fig. 2b), but also an enhanced *MART-1* expression as demonstrated by both RT-PCR (Fig. 2c) and immunostaining (Fig. 2d). Using these GFP^high^ H1.MP cells, we derived DCs (GFP^high^ H1.MP-DCs) and checked their tumour antigen presentation. As shown in Fig. 2e, these DCs were capable of expanding primed MART-1-specific CD8+ T cells in a restimulation process. However, we also observed that these DCs were not efficient for T cell priming (data not shown). These results suggest that tumour antigen is expressed, processed and presented by GFP^high^ H1.MP-DCs, but the antigen presentation level may not be sufficient to prime a T cell response.

### Modification of hPSCs with tumour antigen epitope-coding minigene

**Figure 3:**
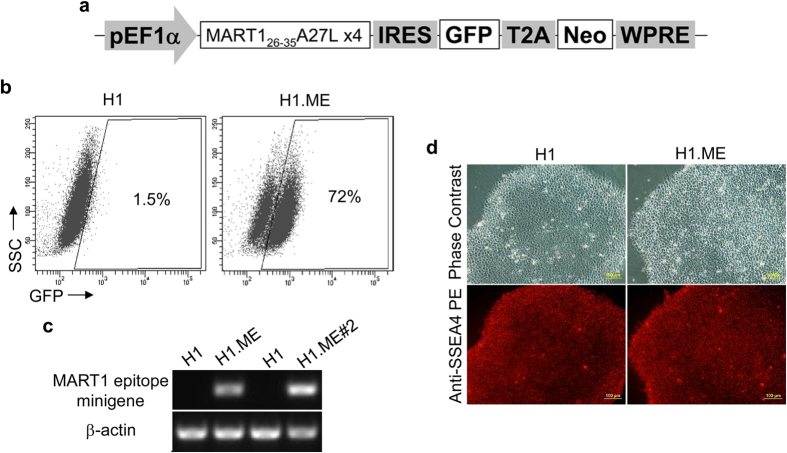
Modification of hPSCs with tumour antigen epitope-coding minigene. (**a**) Structure of lentivector LV.ME carrying MART-1 epitope-coding minigene. (**b**) GPF expression in H1.ME cells, a H1 cell line generated by LV.ME transduction and G418 selection, as detected by flow cytometry. (**c**) Minigene expression in H1.ME cells as measured by RT-PCR. (**d**) SSEA-4 expression in H1.ME as detected by immunostaining.

In addition to tumour antigen expression level, efficient antigen processing is equally crucial for antigen presentation on DC surface. It is well studied that some tumour antigens including MART-1 are poorly processed by immunoproteasomes of DCs^12^. To facilitate MART-1 antigen processing and thus to enhance its presentation on hPSC-DCs, we generated another lentivector, LV.ME to antigenically modify H1 cells (Fig. 3a). Instead of carrying the whole *MART-1* gene, this LV.ME was carrying a synthetic minigene composed of an ubiquitin sequence for proteasomal targeting and a sequence of four repeats of a MART-1 epitope to facilitate antigen processing as well as to increase the copy number of antigenic epitope. This lentivector was able to efficiently modify H1 cells as demonstrated by significant GFP expression in a resulting cell line, H1.ME (Fig. 3b). RT-PCR result showed that the minigene was also expressed in H1.ME cells (Fig. 3c). Furthermore, such genetic modification using minigene did not affect “stem cell” status as indicated by the typical hPSC morphology and SSEA-4 expression in H1.ME cells (Fig. 3d).

### Tumour antigen epitope-coding minigene is expressed in DCs derived from minigene-modified hPSCs

**Figure 4:**
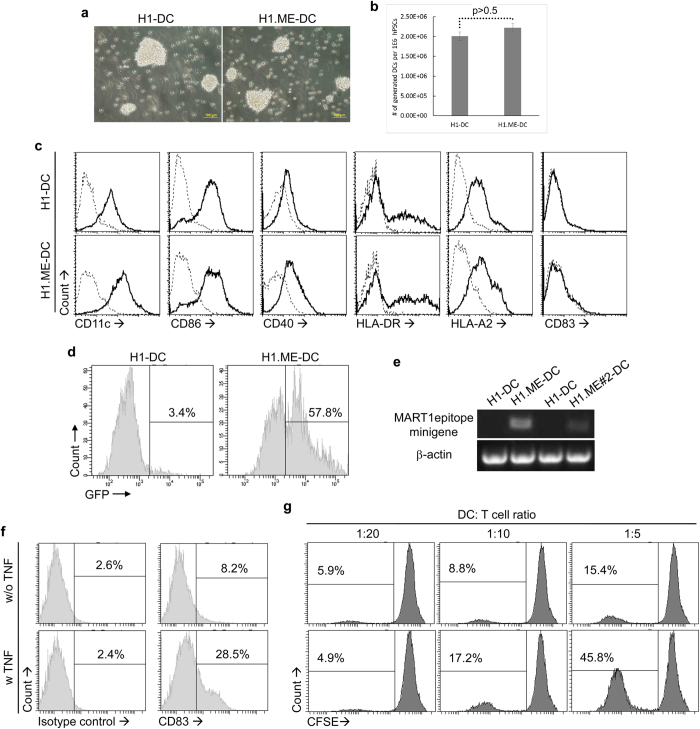
Tumour antigen epitope-coding minigene is expressed in DCs derived from minigene-modified hPSCs. (**a–c**) Morphology (**a**), yield (**b**) and phenotype (**c**) of DCs derived from minigene-modified hPSCs (H1.ME-DCs). The statistical significance of difference was determined by two-sided Student’s t-test (mean ± SD, n = 10) in (**b**). (**d**) GFP expression in H1.ME-DCs as detected by flow cytometry. (**e**) Expression of MART-1 epitope-coding minigene in H1.ME-DCs as measured by RT-PCR. (**f**) CD83 expression on H1.ME-DCs after treatment with TNF. (**g**) Allostimulatory function of H1.ME-DCs on CD4+ T cells after treatment with TNF. The percentages of divided CD4+ T cells are indicated.

To investigate whether the minigene-modified hPSCs can still generate DCs and moreover, whether such generated DCs still express the tumour antigen epitope-coding minigene, we derived DCs from H1.ME cells (H1.ME-DCs) using a three-step protocol as previously described^9^,^10^,^13^. The resulting H1.ME-DCs were similar in morphology (Fig. 4a) and phenotype (Fig. 4c) to those unmodified H1 cell-derived DCs, H1-DCs. They expressed typical DC surface markers like CD11c, CD86, CD40 and HLA-DR, but little CD83 (Fig. 4c), which suggests an immature DC phenotype. They also expressed HLA-A2 (Fig. 4c), a MHC class I molecule that is important for MART-1 epitope presentation in this study. The yield of DCs from H1.ME cells also resembled that from H1 cells (Fig. 4b). In terms of transgene expression, more than half of these H1.ME-DCs remained GFP+ as measured by flow cytometry (Fig. 4d); more importantly, obvious minigene expression was detected by RT-PCR in H1.ME-DCs (Fig. 4e). These results suggest that the minigene-modified hPSCs are able to differentiate into minigene-expressing DCs. For further maturation, H1.ME-DCs were cultured with 20 ng/ml TNF for one day. This TNF-treatment up-regulated the CD83 expression on H1.ME-DCs (Fig. 4f) and improved their allostimulatory function on CD4+ T cells (Fig. 4g), suggesting the immunogenic property of these DCs.

### DCs derived from minigene-modified hPSCs efficiently prime tumour antigen-specific T cell response

**Figure 5:**
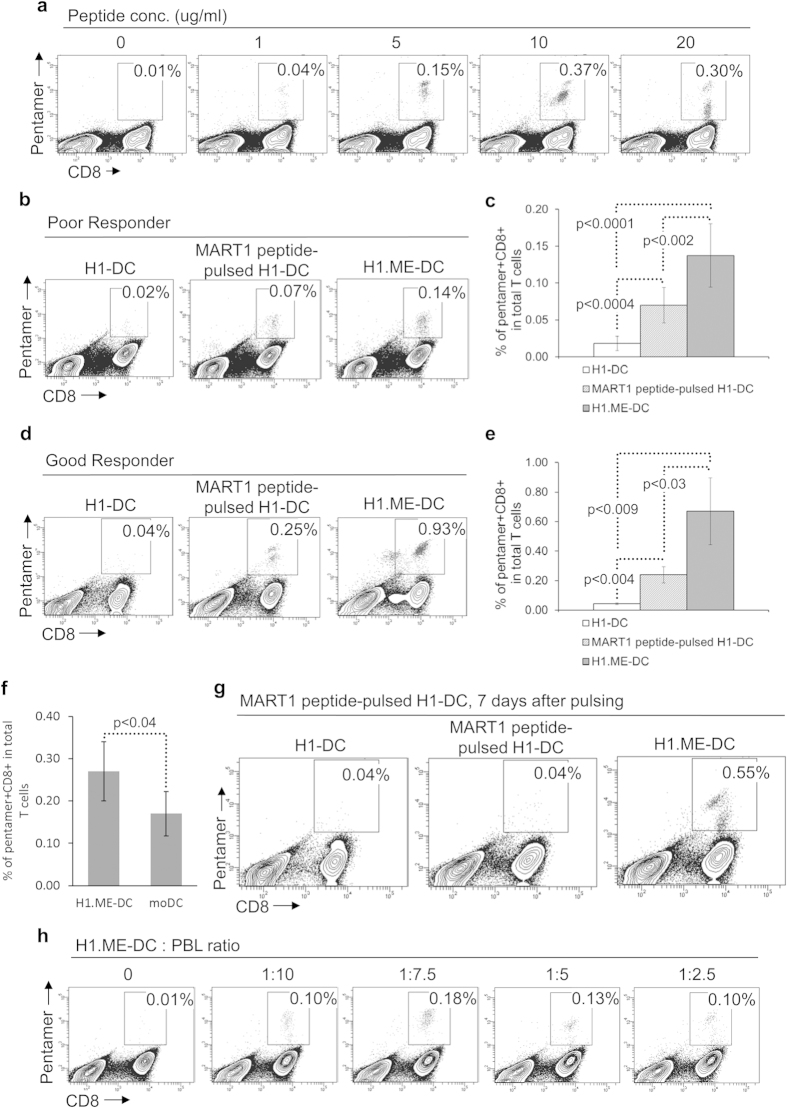
DCs derived from minigene-modified hPSCs efficiently prime tumour antigen-specific T cell response. (**a**) Induction of MART-1-specific CD8+ T cell response by H1-DCs pulsed with MART-1 peptide of various concentrations. (**b,c**) Induction of MART-1-specific CD8+ T cell response by H1.ME-DCs in low-responsive PBLs. The antigen-specific T cells were stained by pentamer and detected by flow cytometry nine days after DC/PBL coculture. (**b**) Contour plots of a representative experiment. The numbers in plots indicate the percentage of pentamer+ CD8+ cells in total T cells. (**c**) Quantitative analysis of the experiments. The statistical significance of differences were determined by two-sided Student’s t-test (mean ± SD, n = 6). (**d,e**) Induction of MART-1-specific CD8+ T cell response by H1.ME-DCs in high-responsive PBLs. (**f**) Comparing T cell priming ability of H1.ME-DCs and MART-1 peptide-pulsed moDCs. The statistical significance of difference was determined by two-sided Student’s t-test (mean ± SD, n = 5). (**g**) Comparing T cell priming ability of MART-1 peptide-pulsed H1-DCs and H1.ME-DCs after prolonged culture. H1-DCs were pulsed with MART-1 peptide, washed and further cultured for seven days before applying for priming. Unpulsed H1-DCs and H1.ME-DCs were employed as controls. (**h**) Induction of MART-1-specific CD8+ T cell response by H1.ME-DCs using different DC:PBL ratios.

To examine whether the expression products of tumor antigen epitope-coding minigene can be efficiently processed and presented in DCs derived from minigene-modified hPSCs, we assessed the ability of H1.ME-DCs to prime a MART-1-specific CD8+ T cell response and compared its efficacy to that of H1-DCs pulsed with 10 μg/ml MART-1 peptide, which is an optimal peptide concentration to load H1-DCs (Fig. 5a). H1.ME-DCs were cocultured with HLA-A2+ peripheral blood lymphocytes (PBLs) from healthy donors. After nine days, MART-1-specific T cells were identified by pentamer staining. As shown with low-responsive PBLs, H1.ME-DCs efficiently primed a MART-1-specific T cell response and the efficacy was significantly better than that of MART-1 peptide-pulsed H1-DCs, which were prepared with a commonly used antigen-loading approach (Fig. 5b,c). Similar results were obtained using high-responsive PBLs (Fig. 5d,e), which further confirmed that the minigene products were efficiently processed in H1.ME-DCs and the resulting tumour antigen epitopes were sufficiently presented on H1.ME-DCs for T cell priming. Moreover, such produced H1.ME-DCs were more efficient than the commonly used moDCs pulsed with MART-1 peptide (Fig. 5f).

To understand this advantage of H1.ME-DCs over MART-1 peptide-pulsed H1-DCs in T cell priming, the sustainability of MART-1 epitope presentation in these two types of DCs were compared side by side. After a 4-hour peptide-pulsing, MART-1 peptide-pulsed H1-DCs were washed and further cultured for 7 days before use for priming. Unpulsed H1-DCs and H1.ME-DCs were employed as controls. After this prolonged culture, MART-1 peptide-pulsed H1-DCs no longer induced a specific T cell response; in contrast, H1.ME-DCs remained very competent (Fig. 5g). This result indicates that H1.ME-DCs have more sustainable MART-1 antigen presentation than MART-1 peptide-pulsed H1-DCs, wherein the formers are continuously supplied with MART-1 epitopes from minigene expression. To explore the possible benefit of using high dosage of DCs in T cell priming, cocultures were set up using H1.ME-DCs and HLA-A2+ PBLs at various DC:PBL ratios (Fig. 5h). The results showed that H1.ME-DCs were able to induce a specific T cell response in a wide range of DC:PBL ratio; yet a ratio higher than 1:5 was not helpful.

### CTLs expanded by DCs derived from minigene-modified hPSCs are immunocompetent

**Figure 6:**
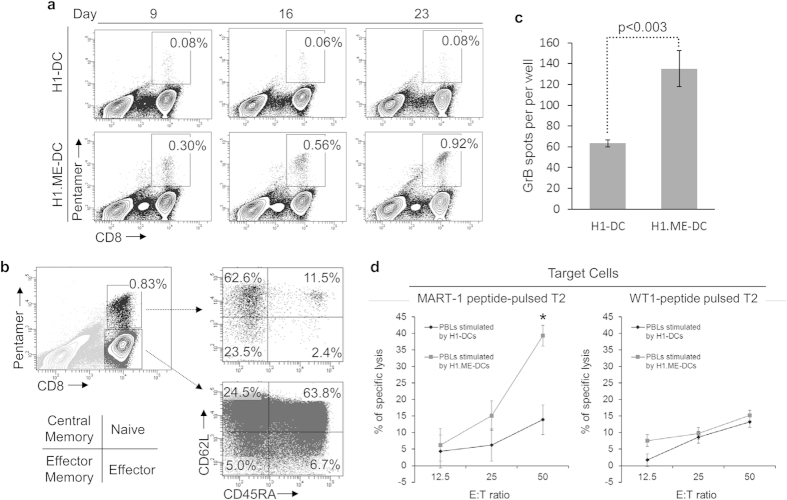
CTLs expanded by DCs derived from minigene-modified hPSCs are immunocompetent. (**a**) Expansion of MART-1-specific CD8+ T cells by H1.ME-DCs in bulk culture. HLA-A2+ PBLs were primed and then restimulated twice with H1.ME-DCs. MART-1-specific T cell expansion during this process was monitored by flow cytometry at the indicated time points. The percentages of pentamer+ CD8+ cells in total T cells are shown in the representative contour plots. (**b**) Phenotype of MART-1-specific T cells expanded by H1.ME-DCs. (**c**) GrB secretion by MART-1-specific T cells expanded by H1.ME-DCs as measured by ELISPOT. The statistical significance of difference was determined by two-sided Student’s t-test (mean ± SD, n = 3). (**d**) Specific cytotoxicity of MART-1-specific T cells expanded by H1.ME-DCs. The statistical significance of difference was determined by two-sided Student’s t-test (mean ± SD, n = 3, *p < 0.002).

To test whether H1.ME-DCs were able to expand antigen-specific cytotoxic T lymphocytes (CTLs), HLA-A2+ PBLs were primed and then restimulated twice with H1.ME-DCs. MART-1-specific T cell expansion during this process was monitored by pentamer staining. As shown in Fig. 6a, the MART-1-specific T cell population increased with each stimulation by H1.ME-DCs, but not by H1-DCs. Interestingly, these expanded MART-1-specific CTLs predominantly possessed central memory or effector memory phenotype (Fig. 6b), which correlate with the less differentiated T cell populations that have better anti-tumour immunity. To test the function of these expanded CTLs, the secretion of granzyme B (GrB) was detected by ELISPOT. As shown in Fig. 6c, the CTLs expanded by H1.ME-DCs were responsive to stimulation by MART-1 peptide-pulsed T2 cells. Furthermore, these CTLs were not functionally exhausted after multiple stimulations. They were able to specifically kill target cells as demonstrated by cytotoxicity assay (Fig. 6d). These results suggest that H1.ME-DCs are competent antigen-presenting cells for specific CTL expansion.

## Discussion

DC vaccine is a major strategy in cancer immunotherapy^14^. In the age of immunotherapy and pluripotency, generating DCs from hPSCs provides a promising and attractive platform technology for centralized and large-scale DC vaccine production^8^,^9^,^10^,^13^,^15^,^16^,^17^,^18^. To develop clinically successful and commercially viable DC products from hPSCs, it is necessary to redesign, optimize and simplify the production process of DC vaccine accordingly. By exploiting the amenability of hPSCs to genetic modification, we have demonstrated in this study a unique tumour antigen-loading strategy that may simplify the hPSC-DC vaccine production. Specifically, we showed that antigenically modified hPSCs were able to differentiate into tumour antigen-presenting DCs. Without any manipulations of conventional antigen-loading, these antigen-loaded hPSC-DCs were ready to prime a tumour antigen-specific T cell response and further expand these specific T cells in restimulation processes. Such expanded T cells were immunocompetent antigen-specific effectors with central memory or effector memory phenotype.

In DC vaccine production, tumour antigen-loading is one of the most critical steps. The choice of tumour antigen-loading approach determines how the antigen is processed and presented by DCs and thus determines the ensuing anti-tumour immune response. For cancer vaccine, induction of tumour antigen-specific CTL responses that directly kill tumour cells remains a primary task. Thus, it is important to control the antigen presentation pathway utilized by the loaded tumour antigens, preferably an endogenous pathway in the case of cancer vaccine, wherein tumour antigens are presented by MHC class I to CD8+ T cells. Currently, several antigen-loading approaches have been used in DC vaccine production. Protein- or tumour lysate-loading approach provides the possibility to present multiple antigenic epitopes without MHC-restriction, but this approach requires a large amount of expansive clinical-grade tumour antigen protein or tumour cell lysate. Moreover, the loaded tumour antigens tend to be presented by MHC class II rather than MHC class I^19^. Peptide-pulsing is a simple approach to load DCs with tumour antigen for presentation to CD8+ T cells, in which the MHC-restricted tumour antigenic peptides bind directly to MHC class I without going through the antigen processing pathways. However, like other exogenous antigen-dependent approaches, peptide-pulsing only provides short antigen presentation duration due to the high turnover rate of MHC/peptide complexes^20^. Nucleic acid-based antigen-loading approach may extend the duration of tumour antigen presentation in DCs. In this approach, tumour antigen-coding DNA or RNA are delivered into DCs and the expression of these nucleic acids may provide an endogenous supply of cytosolic tumour antigens that incline to be presented via endogenous pathway^21^. However, the antigen presentation efficiency of such approach depends on efficient transgene expression in DCs. For DNA-based antigen-loading, viral vectors such as adenoviral, retroviral and lentiviral vectors are preferably used than other nonviral methods due to a higher level of transgene expression in DCs^22^. However, generation of clinical-grade viral vectors and clinical use of these vectors are still very challenging. In contrast, RNA-based antigen-loading does not depend on viral vector, tumour antigen-coding RNA can be delivered via electroporation into DC cytoplasm, wherein RNA is translated to produce tumour antigens. Unlike the DNA-based approach, RNA-based approach does not require a transcription step, thus it is more efficient. However, the antigen presentation duration is limited by the poor stability and short lifespan of RNA^23^.

From the standpoint of DC vaccine production, all the above-mentioned conventional antigen-loading approaches require the production of clinical-grade antigen payloads in various forms, such as peptides, proteins, tumour cell lysates, DNA or RNA. Additional manipulations on DCs are mandatory to deliver these antigen payloads into DCs for the subsequent presentation on DC surface. Such manipulations including cell incubation, transfection, electroporation and viral transduction would more or less reduce the yield and viability of DC vaccine and have to be repeated for every batch of DC product. Moreover, antigen-loading is not a stand-alone step. It needs to be coordinated with *ex vivo* DC generation and maturation steps, which further complicates the whole production process of DC vaccine. Thus, to streamline DC vaccine production from hPSCs, it is highly recommended to avoid all these DC-oriented conventional antigen-loading approaches and come up with a different solution that suits this novel hPSC-DC platform. As demonstrated in this study, unlike in a patient blood cell-dependent platform, in which antigen-loading is restricted to DCs, antigen-loading in an hPSC-DC platform can be done in hPSCs other than DCs.

On top of providing unlimited and quality-controlled DCs, generating DCs from hPSCs has another major advantage for DC vaccine production: hPSCs are very amenable to genetic modification^24^. Previously, we have demonstrated that a transgene such as *CD1d* that is stably integrated into hPSC genome retains its expression in the DCs derived from such modified hPSCs^10^. Here we show that this is also applicable to tumour antigen genes in the form of either a full-length sequence or an artificial minigene. Most importantly, the expression products of these tumour antigen genes are further processed and presented by hPSC-DCs, which efficiently induce an antigen-specific CD8+ T cell response. This observation implies that DCs generated from antigenically modified hPSCs are already antigen-loaded. There are several advantages using this novel antigen-loading strategy for DC vaccine production from hPSCs: (1) It streamlines the production process by totally eliminating the DC-restricted conventional antigen-loading step. There are no more requirements of clinical-grade payload production and additional DC manipulation. Since the antigen-loading is done in hPSCs, the logistic issue of whether to load DCs before or after maturation also becomes irrelevant. (2) It enhances DC vaccine efficacy. In terms of antigen presentation pathway, tumour antigens are synthesized endogenously from the integrated transgene, thus naturally channeled to endogenous pathway for presentation by MHC class I, which is preferable for cancer vaccine. With respect to antigen presentation duration, the continuous supply of tumour antigens by constitutive expression of the integrated transgene ensures persistence of antigen presentation on hPSC-DCs, which is critical for DC immunogenicity. (3) It may facilitate further development of hPSC-DC vaccine. Antigenically modified hPSCs can provide an unlimited amount of standardized tumour antigen-loaded DCs for optimizing other important procedures such as DC generation, DC maturation and DC cryopreservation. (4) It may provide the possibility to present both MHC class I- and MHC class II-restricted epitopes in DC vaccine. It is well-known that CD4+ helper T cells also contribute to anti-tumour immunity by activating DCs and by producing optimal cytokines^25^. DC vaccines that activate CD4+ helper T cells simultaneously may be useful to further improve tumour antigen-specific CTL response. Although this study has been focusing on antigen presentation to CD8+ T cells, by using a synthetic transgene that includes HLA class II-restricted epitopes, this antigen-loading strategy may also be applied for presenting antigens to CD4+ T cells.

Apparently, the successful translation of this novel antigen-loading strategy not only depends on our in-depth understanding of DC biology, but also the overall development of hPSC application. With the advances in induced pluripotent stem cell (iPSC) technology, the derivation of clinical-grade patient-specific iPSCs will eventually become less costly and less time-consuming. Such iPSCs may also be pre-made and stored to provide fully HLA-matched iPSCs that can be used to generate antigen-presenting DCs for an autologous setting. Before that, DCs generated from allogeneic iPSC lines expressing the common HLA haplotypes may be enough to fulfil the purpose of antigen presentation. Unlike in the setting of regenerative medicine, wherein HLA-matched transplant is required for long-term engraftment, the requirement of histocompatibility in DC-based therapy is less stringent since long-term survival of DCs is not necessary. However, in such allogeneic setting, it is crucial for DCs to initiate anti-tumour responses quickly and effectively before their elimination by allo-reactive CTLs of the recipient. Another challenge for our antigen-loading approach is the modification of hPSCs. As a proof-of-principle, we have used lentiviral vector to deliver tumour antigen genes in this study. Although lentiviral vector has been used in clinical studies^26^, other safer genome engineering approaches, such as Zinc-finger nucleases (ZFNs), transcription activator-like effector nucleases (TALENs) and clustered regulatory interspaced short palindromic repeat (CRISPR)/Cas-based RNA-guided DNA endonucleases, are preferred to integrate a transgene into a “safe harbour”^27^,^28^. Combining together with these technologies, our antigen-loading strategy may further increase the clinical feasibility of hPSC-DC vaccine.

**Figure 7:**
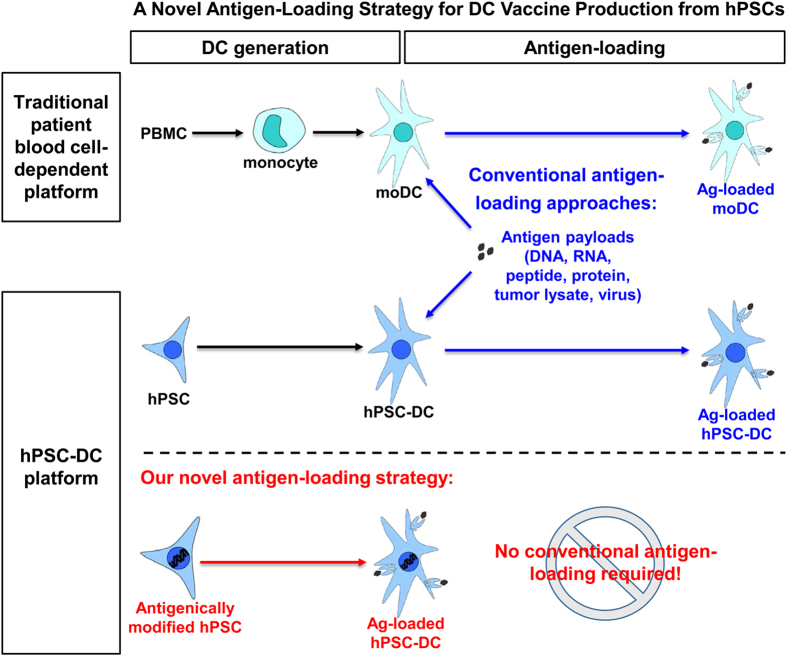
Schematic summary of a novel antigen-loading strategy for DC vaccine production from hPSCs. In a traditional patient blood cell-dependent platform, antigen-loading is limited to DCs, wherein antigen payloads in various forms are delivered into DCs by conventional antigen-loading approaches. In an hPSC-DC platform, antigen-loading can be done in hPSCs other than DCs by antigenically modifying the hPSCs. From such antigenically modified hPSCs, antigen-loaded DCs can be generated without a conventional antigen-loading step. Using this novel antigen-loading strategy, there are no more requirements of clinical-grade payload production and additional DC manipulation. Thus, DC vaccine production from hPSCs is significantly simplified.

In summary (Fig. 7), our study demonstrates that tumour antigen-presenting DCs can be directly generated from antigenically modified hPSCs. With this approach, there is no conventional antigen-loading step required and DC vaccine production from hPSCs is significantly simplified.

## Methods

### Cell culture and DC generation

An hPSC line, H1 (WiCell Research Institute, Madison, WI, http://www.wicell.org), was maintained with a serum-free and feeder-free culture system using mTeSR1 medium (StemCell Technologies, Vancouver, BC, Canada, http://www.stemcell.com) and Matrigel (BD Biosciences, San Diego, CA, http://www.bdbiosciences.com) -coated six-well plates according to manufacturer’s technical manual. OP9 cells [American Type Culture Collection (ATCC), Manassas, VA, http://www.atcc.org] were cultured with α-MEM (Life Technologies, Carlsbad, CA, http://www.lifetechnologies.com) supplemented with 20% fetal bovine serum (FBS) (HyClone, Logan, UT, http://www.hyclone.com). T2 cells (ATCC) were cultured with IMDM (Life Technologies) supplemented with 20% FBS.

To derive human DCs from H1 cells, we used a three-step protocol as described previously^9^,^10^,^13^. In brief, OP9 cells were seeded on 0.1% gelatin (Sigma-Aldrich, St Louis, MO, http://www.sigmaaldrich.com) -coated T75 flask. Upon confluence, the culture were fed by changing half of the medium and overgrown for 4–6 days. 1–1.5 × 10^6^ H1 cells were then seeded and differentiated on the overgrown OP9 cells in α-MEM supplemented with 10% FBS and 100 μM monothioglycerol (Sigma-Aldrich). The coculture were fed on day 4 and 6 by changing half of the medium and harvested on day 9 using 1 mg/ml collagenase IV (Life Technologies) and 0.05% trypsin-0.5 mM EDTA (Life Technologies). The harvested cells were further cultured for 10 days in a poly 2-hydroxyethyl methacrylate (Sigma-Aldrich) -coated T75 flask using α-MEM supplemented with 10% FBS, 100 μM monothioglycerol and 100 ng/ml GM-CSF (Peprotech, Rocky Hill, NJ, http://www.peprotech.com). To generate human DCs, these cells were then purified by density gradient centrifugation using 25% Percoll solution (Sigma-Aldrich) and cultured in StemSpan serum-free expansion medium (StemCell Technologies) supplemented with 1% lipid mixture 1 (Sigma-Aldrich), 100 ng/ml GM-CSF and 100 ng/ml IL-4 (Peprotech) (also known as DC-differentiation medium) for 8–12 days.

To obtain human PBLs, frozen HLA-A2+ PBMCs from healthy donors (StemCell Technologies) were thawed and cultured in complete RPMI 1640 medium, which contains RPMI 1640 (Life Technologies) supplemented with 10% heat-inactivated human serum AB (Gemini Bio-Products, West Sacramento, CA, http://www.gembio.com), 2 mM L-glutamine (Life Technologies), 0.1 mM nonessential amino acids (Life Technologies), and 0.1 mM 2-mercaptoethanol (Life Technologies). After 2-hour incubation, the cells in suspension were harvested as PBLs. To derive moDCs, the plastic-adherent cells were differentiated in DC-differentiation medium for 6 days.

### Lentivector preparation and hPSC modification

Two types of lentivectors were generated using two different transfer plasmids. To construct a transfer plasmid carrying a tumour antigen gene *MART-1*, the coding sequence of *MART-1* was cloned from Plasmid MART-1 (ATCC) by PCR to include a Kozak sequence upstream of its start codon and EcoRI and BamHI restriction sites at its termini. These two sites were used to insert *MART-1* gene into pCDH-EF1-MCS-IRES-coGFP-Neo (System Biosciences, Mountain View, CA, http://www.systembio.com). To construct another transfer plasmid carrying a transgene encoding four repeats of a HLA-A2-restricted MART-1 epitope (MART-1_26–35_A27L, ELAGIGILTV), a minigene was synthesized (1st BASE, Singapore, http://www.base-asia.com). This minigene encodes the following amino acid sequence: *MQIFVKTLTGKTITLEVEPSDTIENVKAKIQDKEGIPPDQQRLIFAGKQLEGRTLSDYNIQKESTLHLVLRLRG*VVNSEFKHE**ELAGIGILTV**AEFKSE**ELAGIGILTV**AEE**ELAGIGILTV**AEE**ELAGIGILTV**AEEVNRA, wherein an ubiquitin sequence (italic and underlined) was placed before the sequence of four MART-1 epitopes (bold and underlined) for proteasomal targeting and the codon usage was optimized for expression in human cells. The minigene was cloned and inserted into pCDH-EF1-MCS-IRES-coGFP-Neo using NheI and BamHI sites. Lentivectors, named LV.MP and LV.ME, were produced by contransfecting 293FT cells (Life Technologies) using the above-described constructs and packaging plasmids (System Biosciences). Virus titers were determined using 293FT by transduction with virus after serial dilution and subsequent antibiotic selection.

To genetically modify H1 cells, H1 cell clumps were seeded at a low cell density on Matrigel-coated six-well plates. Two days later, H1 cells were transduced by incubating with LV.MP or LV.ME at a multiplicity of infection of 10 for 6 hours. Antibiotic selection with 50 μg/ml G418 (Life Technologies) was started 3 days after transduction. The resulting G418-resistant H1 lines, designated as H1.MP or H1.ME, were used to derive DCs, designated as H1.MP-DCs or H1.ME-DCs.

### RT-PCR and immunostaining

To detect *MART-1* gene or minigene expression, total RNA of modified H1 cells or their DC progenies were extracted using TRIzol Reagent (Life Technologies). First-strand cDNA was then synthesized using SuperScript III First-Strand Synthesis System for RT-PCR (Life Technologies). One μl of cDNA reaction mix was used to amplify the whole *MART-1* gene or the minigene using PCR SuperMix (Life Technologies). The PCR products were separated by electrophoresis in 1% agarose gel.

To detect MART-1 protein expression, the modified H1 cells were fixed with 4% paraformaldehyde (Sigma-Aldrich) and incubated with a primary antibody against MART-1 (Santa Cruz Biotechnology, Dallas, TX, http://www.scbt.com) for one hour. After washing, a secondary antibody goat anti-mouse IgG-TR (Santa Cruz Biotechnology) was used for visualization under fluorescence microscope.

### Priming, expansion and detection of tumour antigen-specific T cells

To prime a tumour antigen-specific T cell response, 1 × 10^5^ modified H1-derived DCs were matured with 20 ng/ml TNF (Peprotech) for one day and then cocultured with 1 × 10^6^ HLA-A2+ PBLs in complete RPMI medium. Unpulsed H1-DCs and H1-DCs pulsed by 10 μg/ml MART-1 peptide (MART-126-35A27L, ELAGIGILTV; ProImmune, Oxford, U.K., http://proimmune.com/ecommerce/index.php) for 4 hours were also used as negative and positive controls, respectively. Nine days after coculture, the samples were stained with APC mouse anti-human CD3 (BD Biosciences), FITC-labelled anti-CD8 (ProImmune) and R-PE-labelled A*0201/ELAGIGILTV Pentamer (ProImmune). MART-1-specific CD8+ T cells were detected using a FACSAria flow cytometer (BD Biosciences).

To expand MART-1-specific CD8+ T cells, bulk cultures were started with 2 × 10^6^ H1.ME-DCs and 20 × 10^6^ PBLs. After incubation of 9 days, the cells were restimulated twice on a weekly basis with H1.ME-DCs at DC:PBL ratio of 1:10. Bulk cultures stimulated with H1-DCs were used as controls. MART-1-specific CD8+ T cells in the bulk cultures were stained and monitored by FACSAria flow cytometer.

### Flow cytometry and allostimulation assay

To study the phenotype of H1.ME-DCs, the cells were stained with antibodies against CD11c, CD40, CD83, CD86, HLA-DR and HLA-A2 (BD Biosciences) and analyzed with a FACSCalibur flow cytometer (BD Biosciences). To check the phenotype of MART-1-specific CD8+ T cells after multiple stimulations, the cells were stained with R-PE-labelled A*0201/ELAGIGILTV Pentamer and antibodies against CD8, CD45RA and CD62L (BD Biosciences) before analysis using FACSAria flow cytometer.

To measure the allostimulatory function of DCs, frozen human peripheral blood pan-T cells were thawed and labelled with Carboxyfluorescein diacetate succinimidyl ester (CFSE; Life Technologies) as described previously^9^. To set up allostimulation assay, 2 × 10^5^ CFSE-labelled pan-T cells were co-cultured with DCs at various DC:T cell ratios. After 5-day incubation, the samples were stained with APC mouse anti-human CD4 antibody (BD Biosciences) and the CD4+ T cell proliferation was evaluated by CFSE dilution after gating on CD4+ population using FACSAria flow cytometer.

### ELISPOT and cytotoxicity assay

To measure GrB secretion, a Human Granzyme B ELISpot Kit (R&D Systems, Minneapolis, MN, https://www.rndsystems.com) has been used. In brief, 10^5^ expanded T cells and 10^5^ MART-1 peptide-pulsed T2 cells were cocultured on a human GrB microplate for 4 hours. GrB spots were then stained as described in the manufacturer’s manual and counted using an ImmunoSpot Analyzer (CTL, Shaker Heights, OH, http://www.immunospot.com).

To measure cytotoxicity of the expanded MART-1-specific T cells, a flow cytometry-based VITAL-FR assay was employed^29^. In brief, T2 cells stained with CFSE and pulsed with MART-1 peptide were used as specific target cells, while CFSE-stained T2 cells pulsed with HLA-A2-restricted WT1 peptide (WT1_126–134_, RMFPNAPYL; ProImmune) were used as non-specific target cells. T2 cells stained with Far Red DDAO-SE (FR; Life Technologies) and pulsed with gp120 peptide (HIV-1 env gp120_90–98_, KLTPLCVTL; ProImmune) were used as internal control target cells. After multiple stimulations with H1.ME-DCs or H1-DCs, PBLs were cocultured with 4 × 10^4^ target cells and 4 × 10^4^ internal control target cells at the indicated effector: target (E:T) ratios. Cocultures of target cells and internal control target cells without effector cells were used for comparison. After overnight incubation, all samples were assessed by FACSAria flow cytometer and the % of specific lysis at each E:T ratio was calculated as following: % of specific lysis = [1 − (# of target cells/# of internal control target cells)_for an E:T ratio_/(# of target cells/# of internal control target cells)_without effectors_] × 100%.

### Statistics

The statistical significance of differences was determined by two-sided Student’s t-test. A p value of <0.05 was considered to be statistically significant.

## Additional Information

**How to cite this article**: Zeng, J. *et al.* Antigenically Modified Human Pluripotent Stem Cells Generate Antigen-Presenting Dendritic Cells. *Sci. Rep.* **5**, 15262; doi: 10.1038/srep15262 (2015).
